# *SerpinA3N* is a novel hypothalamic gene upregulated by a high-fat diet and leptin in mice

**DOI:** 10.1186/s12263-018-0619-1

**Published:** 2018-11-29

**Authors:** Domenico Sergi, Fiona M. Campbell, Christine Grant, Amanda C. Morris, Eva-Maria Bachmair, Christiane Koch, Fiona H. McLean, Aifric Muller, Nigel Hoggard, Baukje de Roos, Begona Porteiro, Mark V. Boekschoten, Fiona C. McGillicuddy, Darcy Kahn, Phyllis Nicol, Jonas Benzler, Claus-Dieter Mayer, Janice E. Drew, Helen M. Roche, Michael Muller, Ruben Nogueiras, Carlos Dieguez, Alexander Tups, Lynda M. Williams

**Affiliations:** 1 Rowett Institute, University of Aberdeen, Aberdeen, AB25 2ZD UK; 2 Department of Animal Physiology, Faculty of Biology, Philipps University Marburg, Karl-von-Frisch Str. 8, 35043 Marburg, Germany; 3 Centre for Neuroendocrinology and Brain Health Research Centre, Department of Physiology, School of Medical Sciences, University of Otago, Dunedin, 9054 New Zealand; 4 Department of Physiology, University of Santiago de Compostela, 15705 Santiago de Compostela, Spain; 5 CIBER Fisiopatología de la Obesidad y Nutrición (CIBERobn), Madrid, Spain; 6 Nutrition, Metabolism and Genomics Group, Division of Human Nutrition, Wageningen University, Wageningen, The Netherlands; 7 Nutrigenomics Research Group, UCD Conway Institute, University College Dublin, Dublin, Ireland; 8 Biomathematics & Statistics Scotland (BioSS), Rowett Institute, University of Aberdeen, Aberdeen, AB25 2ZD UK; 9 Nutrigenomics and Systems Nutrition Group, Norwich Medical School, University of East Anglia, Norwich, NR4 7UQ UK

**Keywords:** *SerpinA3N*, Hypothalamus, High-fat diet, Leptin

## Abstract

**Background:**

Energy homeostasis is regulated by the hypothalamus but fails when animals are fed a high-fat diet (HFD), and leptin insensitivity and obesity develops. To elucidate the possible mechanisms underlying these effects, a microarray-based transcriptomics approach was used to identify novel genes regulated by HFD and leptin in the mouse hypothalamus.

**Results:**

Mouse global array data identified *serpinA3N* as a novel gene highly upregulated by both a HFD and leptin challenge. In situ hybridisation showed *serpinA3N* expression upregulation by HFD and leptin in all major hypothalamic nuclei in agreement with transcriptomic gene expression data. Immunohistochemistry and studies in the hypothalamic clonal neuronal cell line, mHypoE-N42 (N42), confirmed that alpha 1-antichymotrypsin (α_1_AC), the protein encoded by *serpinA3*, is localised to neurons and revealed that it is secreted into the media. *SerpinA3N* expression in N42 neurons is upregulated by palmitic acid and by leptin, together with *IL-6* and *TNFα*, and all three genes are downregulated by the anti-inflammatory monounsaturated fat, oleic acid. Additionally, palmitate upregulation of *serpinA3* in N42 neurons is blocked by the NFκB inhibitor, BAY11, and the upregulation of *serpinA3N* expression in the hypothalamus by HFD is blunted in IL-1 receptor 1 knockout (*IL-1R1*^*−/−*^) mice.

**Conclusions:**

These data demonstrate that *serpinA3* expression is implicated in nutritionally mediated hypothalamic inflammation.

## Background

Once considered problematic for only the Western, developed world, the burden of obesity today encompasses most countries [1], with shifts in global eating patterns causing detrimental changes in body weight and composition, associated diseases and decreased quality of life [2, 3]. The development of effective treatments for obesity has not been fruitful, with the continued futility of dieting methodologies for sustained weight loss, and gastric surgery being the only current long-term solution [4–6], putting health care systems under an enormous amount of pressure. In order for more effective, preventative measures to be put in place, it is crucial to understand the mechanisms that cause obesity. The overconsumption of energy-dense foods, particularly those high in long-chain saturated fat and sugar, appears to be a primary driving force behind the obesity epidemic [7]. These foods have been shown to have a powerful effect on the hypothalamus in rodent models, resulting in inflammation and astrogliosis that is implicated in diet-induced obesity [8–10].

Energy balance, food intake and consequently body weight are effectively regulated by a well-defined and complex hypothalamic system. Briefly, two distinct types of neurons have been identified as important in energy balance regulation: orexigenic neurons expressing neuropeptide Y (NPY) and agouti-related peptide (AgRP) and anorexigenic neurons expressing proopiomelanocortin (POMC) and cocaine- and amphetamine-regulated transcript (CART). Both orexigenic and anorexigenic neurons express leptin receptors, highlighting the importance of this hormones in the system. Leptin acts on multiple levels in hypothalamic neurons, effecting gene transcription, post-translational processing as well as membrane polarisation to inhibit AgRP/NPY neurons while activating POMC/CART neurons, resulting in a stable body weight [11]. In the obese state, the ability of leptin to regulate feeding behaviour as well as peripheral glucose homeostasis is diminished as a result of hypothalamic insensitivity to this hormone [12–16].

When animals are fed a high-fat diet (HFD), they ingest more energy than they expend, resulting in increased adiposity, and eventually obesity, suggesting that a HFD compromises the hypothalamic systems that regulate food intake. This has been confirmed in a number of rodent studies showing that a HFD induces inflammation in the hypothalamus activating microglia, astrocytes and neurons via a Toll-like receptor 4 (TLR4)-dependent mechanism [17–19] and the IKKβ/NFκB and JNK inflammatory pathways [20–24] resulting in leptin and insulin insensitivity. Blocking or inhibiting inflammation in the hypothalamus prevents leptin and insulin insensitivity and obesity induced by a HFD [22, 23, 25, 26].

In order to further explore the mechanisms linking HFD to hypothalamic dysfunction and leptin insensitivity, we carried out a microarray-based transcriptomics experiment to determine gene expression changes in response to a HFD as well as leptin challenge. One of the genes that was considerably upregulated by both leptin and HFD was *serpinA3N*, the gene encoding the anti-protease, alpha-1-antichymotrypsin (α_1_AC). In the periphery, α_1_AC is an acute phase protein produced by hepatocytes [27] and has been recently identified as a marker of reactive astrogliosis in the brain [28]. Increased levels of α_1_AC in the brain are commonly associated with inflammatory conditions such as Alzheimer’s disease [29, 30].

In the present study we identified, using transcriptomics, that the gene, *serpinA3n,* is highly expressed in the hypothalamus and is upregulated by leptin and HFD, a finding confirmed by semi-quantitative in situ hybridisation. Furthermore, we have shown that nutritional factors, as well as time on experiment, play a role in the hypothalamic nuclei-specific regulation of *serpinA3N* expression. As the expression of *serpinA3N* in the periphery has been reported to be dependent on inflammation and IL-1 stimulation [31–34], we tested the effect of HFD on hypothalamic *serpinA3N* expression in IL-1 receptor 1 knockout mice (*IL-1R1*^*−/−*^) and in the hypothalamic clonal neuronal cell line, mHypoE-N42 (N42) which we challenged with oleic and palmitic acid, the latter, with and without blocking the NFκB pathway. In *IL-1R1*^−/−^ mice, HFD challenge failed to upregulate *serpinA3N* expression in contrast to wild-type mice and in N42 cells oleic acid downregulated *serpinA3N* while palmitate upregulated expression which was negated by inhibiting the NFκB pathway, confirming that HFD-induced expression is dependent on inflammation. Also, as increased *serpinA3N* expression in the brain has previously been identified as a marker of astrocyte activation [28], we examined α_1_AC immunoreactivity in the hypothalamus together with that of glial fibrillary acidic protein (GFAP), an astrocyte marker, ionised calcium binding adaptor molecule 1 (Iba1), a microglial marker, and NPY and AgRP, markers of neurons important in energy balance. Immunoreactive α_1_AC cells were larger and dissimilar to astrocytes and microglia having a distinctive neuronal shape and close associations with NPY and AgRP beaded fibres, also pointing to neuronal expression of *serpinA3N*. In addition, using Western blotting, the protein product of *serpinA3N,* α_1_AC, was detected in N42 neurons in much lower levels than that found in the media demonstrating that the protein is secreted. The expression of *serpinA3N* in and the secretion of α_1_AC from these cells confirmed neuronal expression of *serpinA3N*.

## Results

### Transcriptomics

**Fig. 1:**
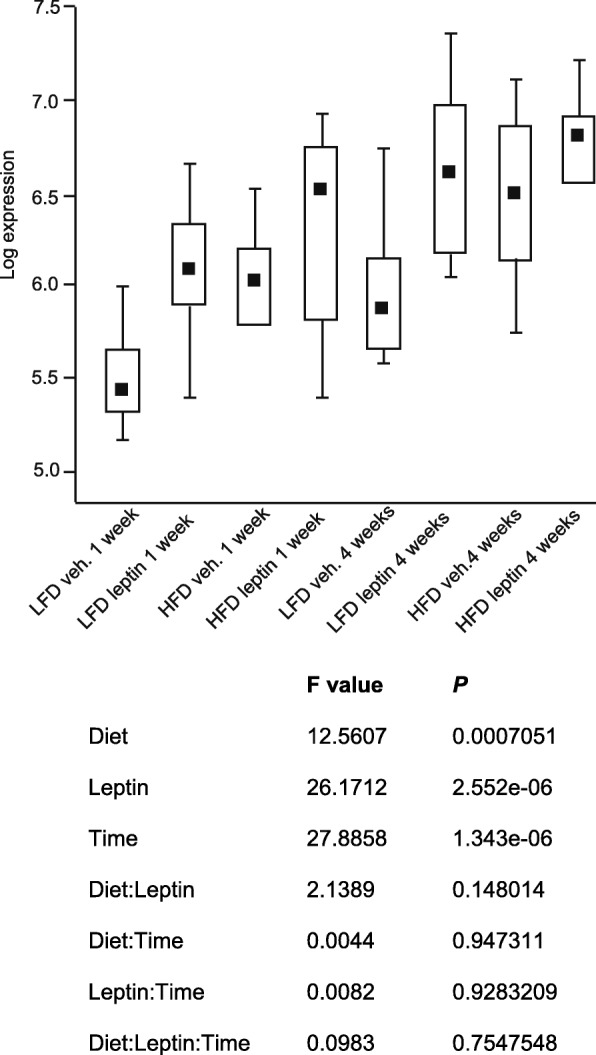
Data derived from NuGO Affymetrics arrays from the hypothalamus of C57BL/6J mice maintained on a HFD or LFD for 1 and 4 weeks challenged with IP vehicle or leptin. The log of *serpinA3N* gene expression is represented by box and whisker plots. Three-way ANOVA showed a significant effect of diet, leptin challenge and time on the diet but no interaction between any of these factors (*n* = 10)

Transcriptomic data revealed the regulation of *serpinA3N* gene expression in total dissected hypothalamic tissue from mice maintained on a LFD or HFD for 1 or 4 weeks and either challenged with vehicle or leptin. A three-way ANOVA showed significant effects of HFD (*P* = 0.0007), leptin challenge (*P* = 2.55 × 10^−6^), and time on diet (*P* = 1.34 × 10^−6^). However, there was no interaction between these factors (Fig. 1). A full list of changed genes can be found at NCBI’s Gene Expression Omnibus and is accessible through GEO Series accession number GSE113943 (https://www.ncbi.nlm.nih.gov/geo/query/acc.cgi).

### Localisation of *serpinA3N* and α_1_AC in the hypothalamus

**Fig. 2:**
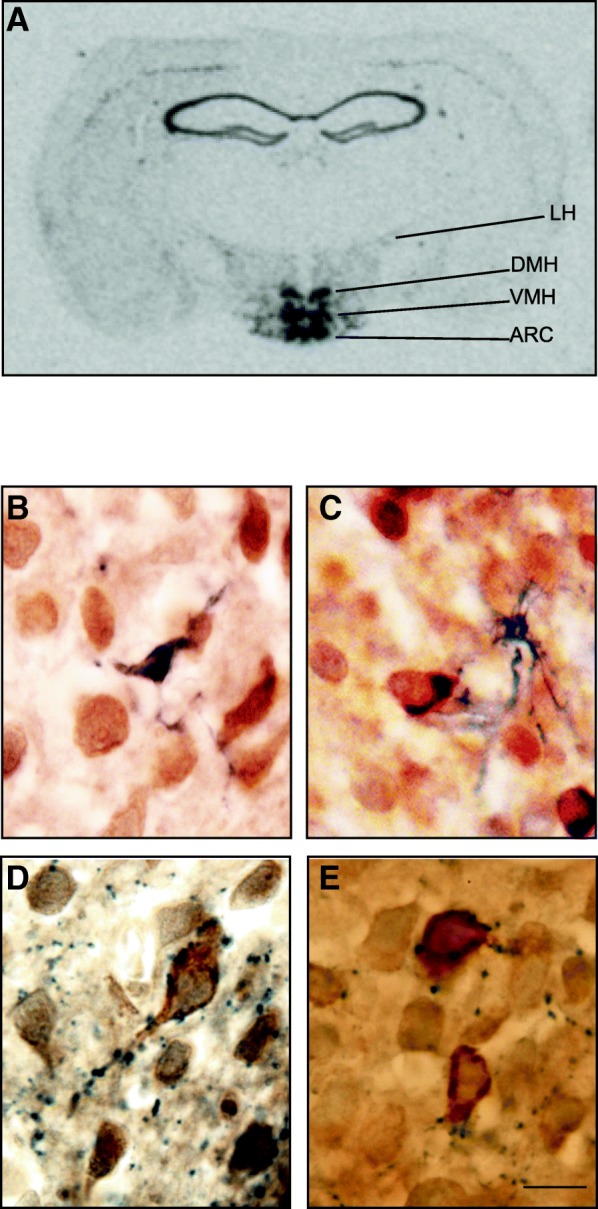
**a** Representative in situ autoradiograph of a C57BL/6J mouse brain section after 4 weeks of HFD showing *serpinA3N* expression in the arcuate nuclei (ARC), the ventromedial nuclei of the hypothalamus (VMH), the dorsomedial nuclei of the hypothalamus (DMH) and the lateral hypothalamus (LH). **b**–**e** Representative immunostaining of α_1_AC (brown) and **b** GFAP, **c** Iba1, **d** AgRP and **e** NPY (all blue) in the arcuate nuclei after 1 week of HFD. Bar = 20 μm

In situ hybridisation using a *serpinA3N*-specific riboprobe revealed specific labelling throughout the hypothalamus with particularly high levels of expression in the arcuate nuclei (ARC), ventromedial (VMH) and dorsomedial nuclei (DMH). The labelling extended out to the edges of the lateral hypothalamus (LH) (Fig. 2a). Immunoreactive α_1_AC in the ARC of the hypothalamus revealed a neuronal morphology (Fig. 2b–e). Dual staining using an anti-mouse α_1_AC antibody (brown) alongside blue anti-GFAP (astrocyte specific) and anti-Iba1 (microglia specific) staining showed smaller and distinctly different cell morphologies with closely associated but not overlapping staining (Fig. 2b, c). Staining with both anti-NPY and anti-AgRP (blue) showed beaded fibres closely associated with anti-α_1_AC-positive neurons (brown) (Fig. 2d, e).

### Regulation of *serpinA3N* and expression by HFD and time

**Fig. 3:**
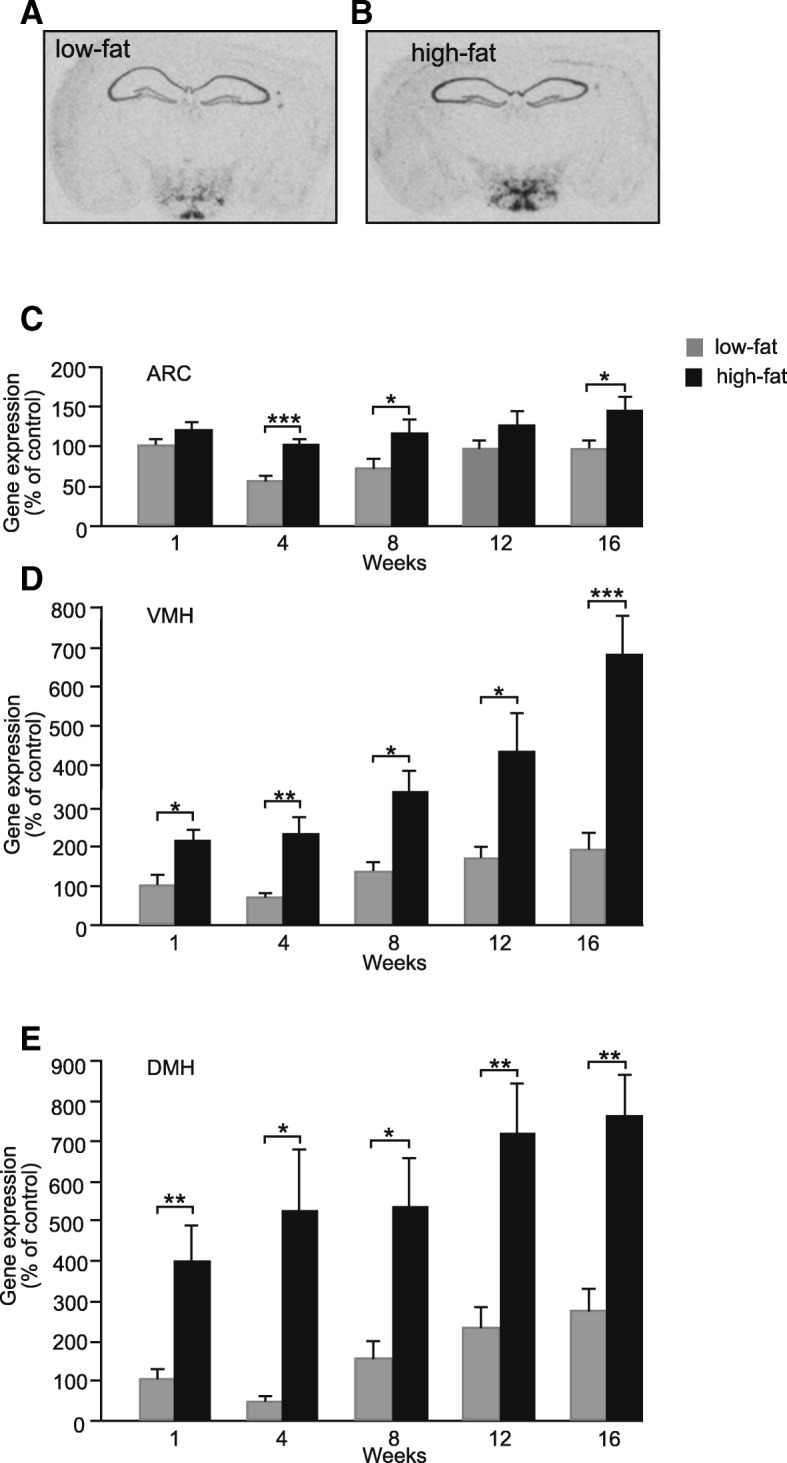
Representative in situ autoradiographs of C57BL/6J mouse brain sections showing *serpinA3N* expression in **a** LFD-fed mouse and **b** HFD-fed mouse. Levels of *serpinA3N* gene expression in LFD- and HFD-fed mice measured by semi-quantitative in situ hybridisation **c** in the arcuate nuclei (ARC), **d** the ventromedial nuclei of the hypothalamus (VMH) and **e** the dorsomedial nuclei of the hypothalamus (DMH). Two-way ANOVA showed a significant effect of diet *P* < 0.001 for the ARC, VMH and DMH and time *P* < 0.001 for the VMH and *P* < 0.05 for the ARC and DMH with an interaction between diet and time *P* < 0.05 for the VMH only. One-way ANOVA showed differences between HFD and LFD from 4 weeks onwards for the ARC and for all times tested for the VMH and DMH. *P < 0.05, **P < 0.01, ***P < 0.001 (*n* = 6)

A robust increase in labelling was seen after just 1 week on a HFD. It was possible to distinguish distinct hypothalamic nuclei which expressed high levels of *serpinA3N* including the ARC, VMH, DMH, and LH (Fig. 3a, b). Quantification of hypothalamic nuclei including the ARC, VMH, DMH (Fig. 3c–e) and LH (not shown) confirmed the increase in *serpinA3N* gene expression by a HFD seen in transcriptomics studies. Expression in the ARC was significantly increased by around 50% after 4 weeks on the HFD compared to LFD control (Fig. 3c). In comparison, the level of gene expression in the VMH and DMH was increased by approximately 2.5 and 4 times after only 1 week, respectively. After 16 weeks on the HFD, the labelling in these nuclei had increased to approximately eight times of that seen in LFD fed mice at week 1 (Fig. 3d, e). Analysis of the gene expression data by two-way ANOVA confirmed that *serpinA3N* gene expression increased with time on the diet and there was no interaction between diet and time for the ARC and DMH but there was for the VMH (*P* < 0.05) indicating that the age of the animals may also lead to increases in *serpinA3N* gene expression in the ARC, VMH and DMH over the 16 weeks of the experiment (Fig. 3c–e).

### Regulation of *serpinA3N* expression by leptin

**Fig. 4:**
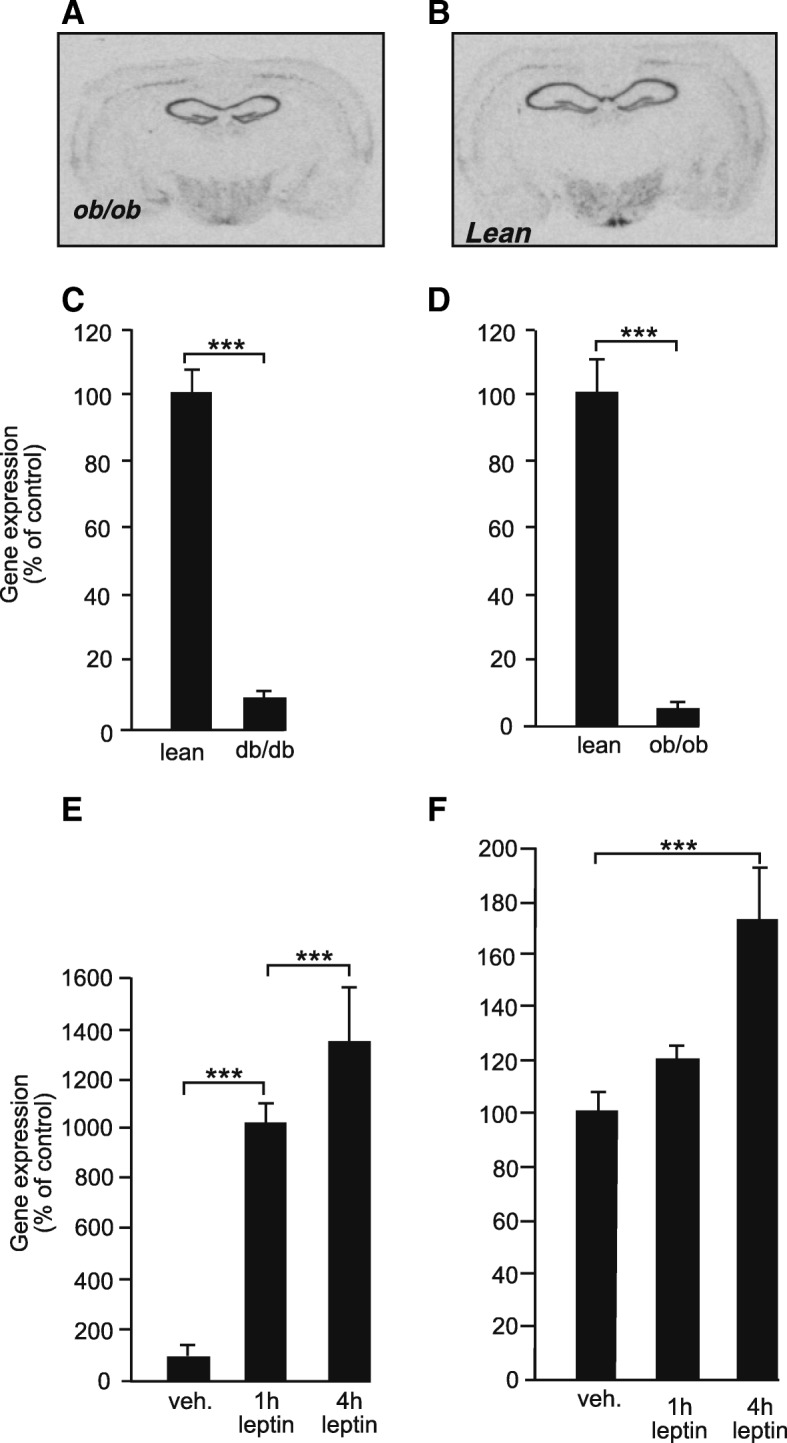
Representative in situ autoradiographs of mouse brain sections showing *serpinA3N* expression in **a** *ob/ob* mouse on a C57BL/6J background and **b** lean C57BL/6J mouse. Levels of *serpinA3N* gene expression in the arcuate nuclei measured by semi-quantitative in situ hybridisation of **c** *db/db* on a C57BL/6J background and lean C57BL/6J mice, **d** *ob/ob* and lean mice, **e** *ob/ob* mice injected with IP vehicle (veh.) or leptin after either 1 or 4 h (h) and **f** lean mice injected with IP vehicle (veh.) or leptin after either 1 or 4 h (h). *** *P* < 0.001 (*n* = 6)

To further investigate the regulation of *serpinA3N* by leptin, in situ hybridisation was carried out on both leptin-deficient mice (*ob/ob*) and mice lacking functional leptin receptors (*db/db*) and their lean littermates (Fig. 4a–d). Images from *ob/ob*, *db/db* and lean mice show a much lower level of *serpinA3N* gene expression in *ob/ob* and *db/db* mice (Fig. 4a, b). Quantification of labelling showed a difference of the order of seven to ten times lower levels of gene expression in *db/db* and *ob/ob* mice compared to their lean littermates (Fig. 4c, d).

Treatment of *ob/ob* mice with leptin (10 mg/ml injected IP) resulted in a time-dependent increase in *serpinA3N* expression with a 10-fold increase within 1 h and a 14-fold increase after 4 h (Fig. 4e). While leptin challenge in lean mice did not increase *serpinA3N* levels of expression above basal after 1 h, leptin challenge did increase levels of expression significantly after 4 h but only by approximately 70% (Fig. 4f) compared to the 14-fold increase seen in *ob/ob* mice 4 h after leptin challenge.

### Regulation of *serpinA3N* expression by HFD in the absence of leptin

**Fig. 5:**
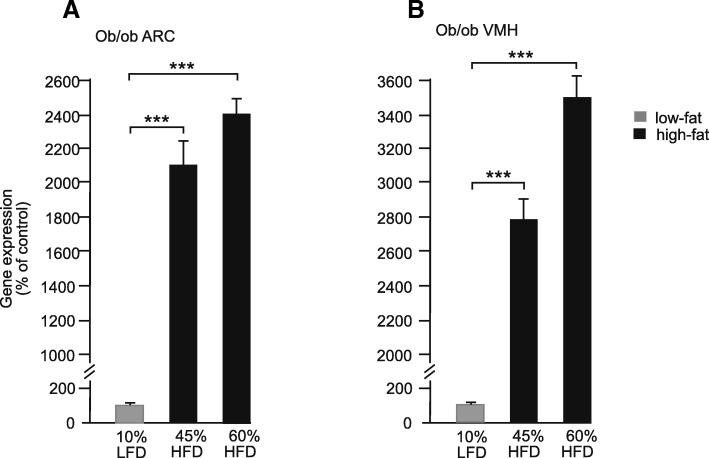
Levels of *serpinA3N* gene expression measured by semi-quantitative in situ hybridisation in the **a** arcuate nuclei (ARC) and **b** ventromedial nuclei of the hypothalamus (VMH) of *ob/ob* mice fed either a LFD, 10% energy (kCal) from fat, or HFD either 45% or 60% energy (kCal) from fat for 8 weeks. ***P < 0.001 (*n* = 6)

Transcriptomics data indicated that the effects of leptin and HFD were independent. To confirm this, *ob/ob* mice, lacking leptin, were fed either a LFD (10% of calories from fat), or a HFD (45% or 60% of calories from fat). The HFD led to a large increase in *serpinA3N* gene expression compared to the LFD in the nuclei expressing the gene. Quantification of labelling showed an increase of around 20–30 times in both the ARC and the VMH after 8 weeks on a HFD (Fig. 5a, b).

### Regulation of *serpinA3N* expression by fasting and refeeding

**Fig. 6:**
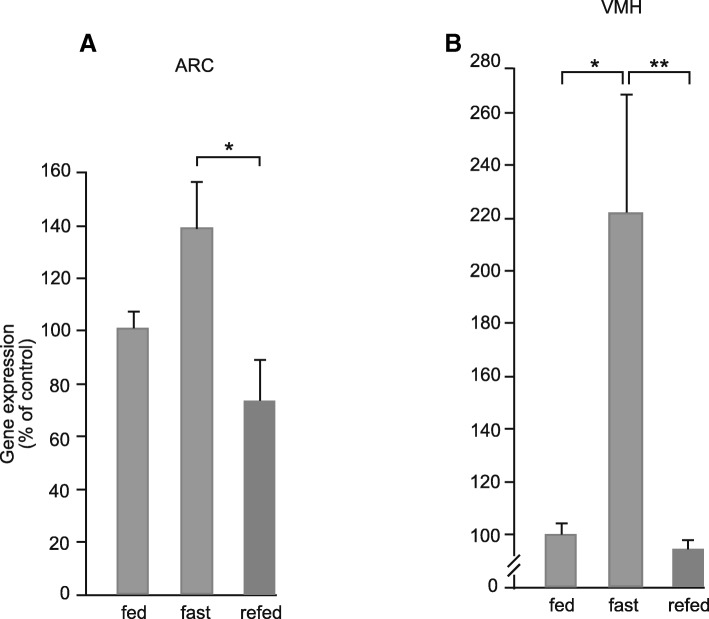
Levels of *serpinA3N* gene expression measure by semi-quantitative in situ autoradiography in the **a** arcuate nuclei (ARC) and **b** ventromedial nuclei of the hypothalamus (VMH) of C57Bl/6J mice either fed, fasted for 24 h or refed for 24 h. *P < 0.05, **P < 0.01 (*n* = 8)

In mice fasted for 24 h, *serpinA3N* expression increased throughout the hypothalamus. Expression increased by around 40% in the ARC and 200% in the VMH. Refeeding the mice for 24 h returned levels of expression to those seen in the fed animals (Fig. 6a, b).

### Regulation of *serpinA3N* by HFD in *IL-1R1*^*−/−*^ mice

**Fig. 7:**
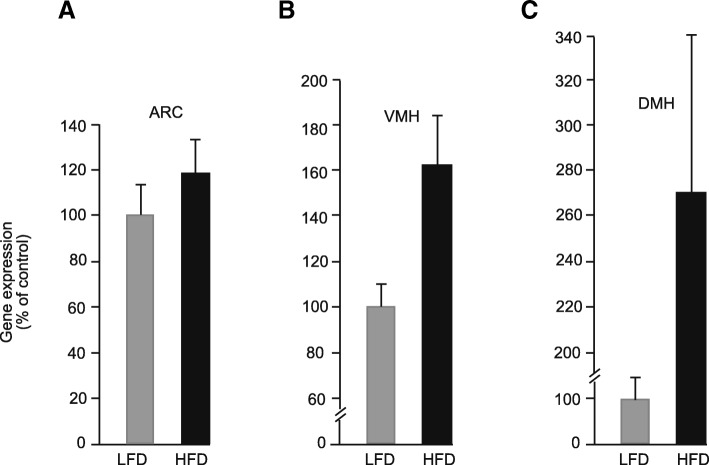
Levels of *serpinA3N* gene expression measured by semi-quantitative in situ autoradiography in the **a** arcuate nuclei (ARC), **b** ventromedial nuclei of the hypothalamus (VMH) and **c** dorsomedial nuclei of the hypothalamus (DMH) in *IL-1R1*^*−/−*^ mice on a C57BL/6J background fed either a LFD or HFD for 8 weeks (*n* = 9 for HFD and *n* = 5 for LFD)

Feeding *IL-1R1*^*−/−*^ mice, a HFD for 8 weeks appeared to increase *serpinA3N* by a relatively small amount in the ARC, VMH and DMH compared to the effects seen in wild-type mice. There was substantial variability in the response to the HFD which failed to reach statistical significance (Fig. 7a–c).

### Regulation of SerpinA3N in N42 cultured hypothalamic neurons

**Fig. 8:**
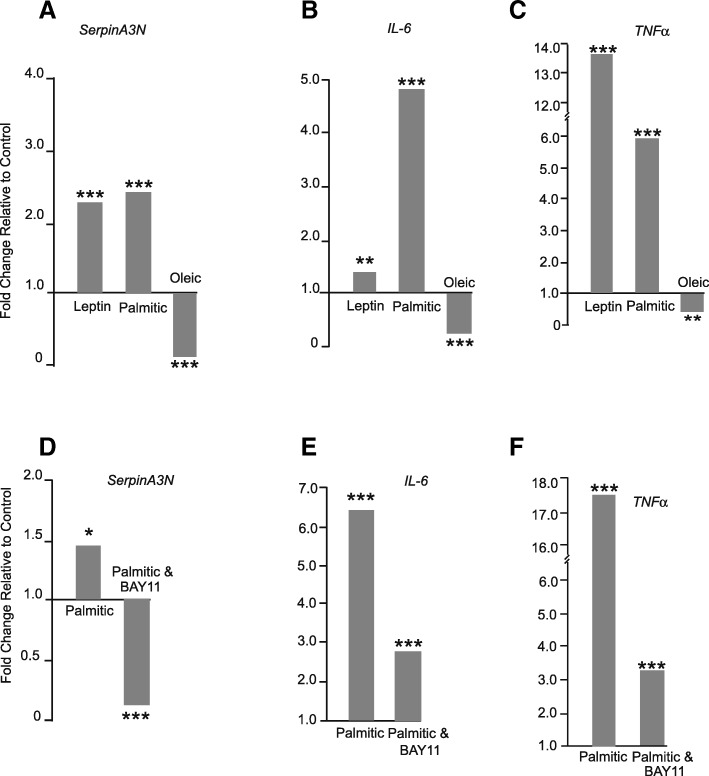
**a**–**f** In mHypoE-N42 cells, a–**c** *SerpinA3N, IL-6 and TNFα* gene expression were all upregulated by leptin and palmitic acid challenge and downregulated by oleic acid. **d**–**f** The upregulation of *SerpinA3N, IL-6 and TNFα* gene expression by palmitic acid was blocked by the NKκB inhibitor BAY11. *P* < 0.05, ***P < 0.001, **P < 0.01. NS not significant

Leptin challenge upregulated *serpinA3N* gene expression by twofold compared to vehicle-treated N42 neurons. *IL-6* and *TNFα* were also upregulated. A similar effect was induced by 200 μM palmitic acid. In comparison, 200 μM oleic acid downregulated the gene expression of all three genes (Fig. 8a–c). The upregulation of *serpinA3N, IL-6* and *TNFα* gene expression by palmitate was significantly reduced in the presence of the NFκB inhibitor, BAY11 (Fig. 8d–f).

### α_1_AC secretion from N42 cultured neurons

**Fig. 9:**
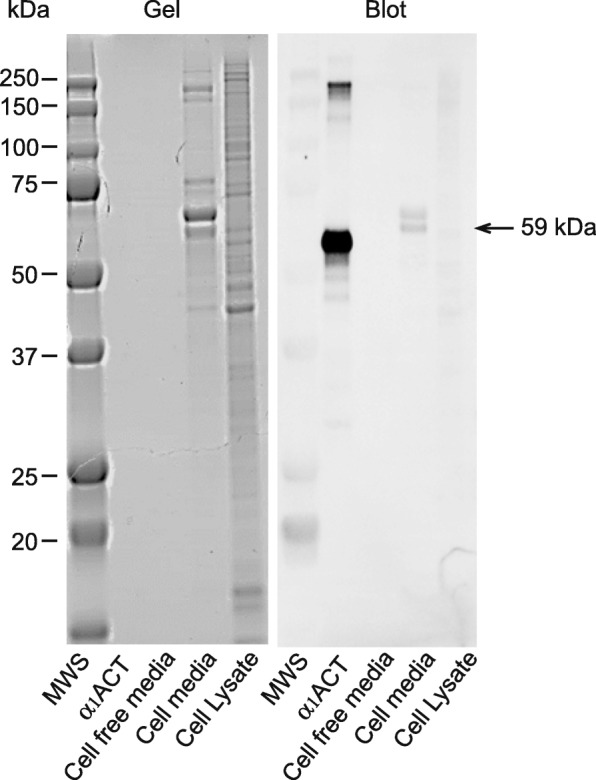
Representative SDS PAGE gel and immunoblot showing alpha-1-antichymotrypsin (α_1_AC), secreted into the media from N42 neuronal cells. No bands are visible with media alone. The blot shows two immunoreactive bands in the media from N42 neurons

SDS-PAGE gels of media from cultured N42 neurons showed the presence of several protein bands of different molecular weights but not in cell free media (Fig. 9). Using Western blotting, cell free media displayed extremely faint immunoreactive α1AC bands but culture media from N42 neurons showed two immunoreactive bands, the lower at 59 kDa corresponding to the molecular weight of α_1_AC (positive control). The band above may correspond to a different post-translationally modified form of the protein. The cell lysate in contrast showed only extremely faint immunoreactive bands including one at 59 kDa (Fig. 9).

## Discussion

Hypothalamic inflammation in response to a HFD in rodents is well-documented after both short- and long-term exposure to HFD [17–19, 35, 36]. In the present study transcriptomics data show that, a so far unreported gene, associated with inflammation in the periphery, *serpinA3N,* is strongly upregulated by HFD and leptin in the hypothalamus. An incidental finding is that *serpinA3N* is also upregulated by time on experiment which corresponds to the increasing age of the mice. Surprisingly, these effects were largely independent of one another indicating that while hypothalamic *serpinA3N* expression is regulated by a number of factors these appear to act via independent pathways.

It can be difficult to differentiate between the effects of a HFD and that of diet-induced obesity as the former rapidly leads to the latter. In the present study, the effects of obesity and a HFD are separated in obese *ob/ob* mice, which, though obese, normally have relatively low levels of hypothalamic *serpinA3N* gene expression, but when challenged with a HFD, the level of expression is rapidly upregulated demonstrating that the level of *serpinA3N* expression is regulated by a HFD rather than obesity per se. Also, the possibility that increased caloric intake rather than diet composition is responsible for the upregulation of *serpinA3N* cannot be ruled out completely in our animal studies. However, *ob/ob* mice are hyperphagic, but as noted above, they express relatively low levels of hypothalamic *serpinA3N* when not exposed to a HFD. Additionally, the pro-inflammatory long-chain saturated fat, palmitic acid, upregulates expression of *serpinA3N* in the clonal neuronal cell line N42, while oleic acid, an anti-inflammatory monounsaturated fatty acid, decreases gene expression indicating that fatty acids have a direct effect on *serpinA3N* expression in neurons, supporting the contention that *serpinA3N* expression responds to the increased long-chain saturated fat in the diet rather than to increased caloric intake.

In the periphery, α_1_AC, the protein encoded by *serpinA3N,* serves as an acute phase protein produced by the liver, in response to inflammation and binds to proteases, thus limiting inflammation-related damage [37, 38]. In the brain, α_1_AC has been shown to be associated with inflammation and elevated levels are found in Alzheimer’s disease, closely linked with amyloid plaques [38–40]. Other observed functions of peripheral α_1_AC include inhibition of oxygen uptake and superoxide generation in granulocytes [41], DNA binding independent of its anti-protease activity, regulation of its own gene expression, and inhibition of DNA polymerase and DNA primase activity [42, 43]. However, the target protein for, and the function of, α_1_AC in the hypothalamus remains to be identified.

In the present study, in situ hybridisation revealed a widespread localisation of *serpinA3N* expression throughout the hypothalamus in a pattern corresponding to major nuclei indicating that the expression is neuronal. This is further supported by immunohistochemistry where dual immunostaining for α_1_AC and both the astrocyte marker, GFAP, and the microglial marker Iba1 reveal no overlap in staining and immunopositive α_1_AC show a characteristic neuronal shape which is both larger and distinctly different from that of GFAP and Iba1 immunopositive cells. Dual staining with NPY and AgRP shows close associations between α_1_AC immunopositive fibres and NPY and AgRP beaded fibres. Also, in N42 clonal neurons in culture, which have been used previously to investigate hypothalamic control mechanisms [44], the expression of *serpinA3N* is upregulated by both leptin and palmitate in a manner consistent with that seen in the hypothalamus in response to leptin challenge and a HFD. Taking all of this evidence together, *serpinA3N* measured in the present study appears largely neuronal in origin. Nonetheless, in a previous genomic study involving two mouse models of brain injury (ischemic stroke and neuroinflammation), *serpinA3N* was identified as a marker of reactive astrogliosis [28], and as HFD has been shown to induce astrogliosis in mice [17, 45], then potentially, some of the increase in *serpinA3N* expression may be astrocytic. Thus, we cannot rule out that part of the increased gene expression seen may occur in astrocytes.

In the liver, increased levels of *serpinA3N* gene expression and subsequently increased levels of α_1_AC, like all acute phase proteins, are stimulated by inflammation, particularly by raised circulating levels of IL-6 and IL-1 type cytokines working via STAT3 activation and increased NFκB [46]. In N42 neurons, both *IL-6* and *TNFα* were upregulated in parallel with *serpinA3N* by leptin and palmitic acid, with palmitic acid stimulated gene expression inhibited by BAY11 blocking the NFκB pathway. These results together with the lack of HFD-induced *serpinA3N* upregulation in *IL-1R1*^−/−^ mice clearly indicated that hypothalamic *serpinA3N* is involved in the inflammatory response as in the periphery.

Leptin stimulates the release of IL-1β in the hypothalamus [47] while a HFD does not appear to upregulate *IL-1β* in this brain area [17] but IL-1β does increase in the periphery in obesity [48] indicating a more complex relationship between HFD, IL-1β and *serpinA3N* upregulation. Circulating interleukins do not readily cross the blood-brain barrier but influence the brain via intermediate mechanisms [49]. The source of the interleukins and the mechanisms underlying upregulation of *serpinA3N* expression by a HFD requires further investigation*.* While there is no significantly different upregulation of *serpinA3N gene* expression in *IL-1R1*^−/−^ mice, there is an indication of a variable response to HFD which may be due to increases in circulating leptin levels in response to increasing adiposity and, thus, increasing leptin levels.

While in the absence of leptin, signalling levels of *serpinA3N* expression in the hypothalamus are low and leptin challenge upregulates *serpinA3N* expression, the localisation and pattern of leptin-stimulated expression differs from that induced by a HFD. Leptin-induced *serpinA3N* expression increases mainly in the ARC, VMH and throughout the hypothalamus up to the borders of the LH with no clear demarcation of hypothalamic nuclei other than the ARC and VMH. The role of leptin in the stimulation of *serpinA3N* expression on a HFD, however, appears to be relatively small as a HFD upregulates *serpinA3N* gene expression to a much greater degree in *ob/ob* mice, in the absence of leptin, compared to lean mice in the presence of leptin. The reasons for this are not clear, but counterintuitively, it may be that the presence of leptin is somehow protective against the induction of *serpinA3N* gene expression by a HFD. Leptin receptors are present on both neurons and astrocytes and leptin may be upregulating *serpinA3N* in either or both these cell types [50], with the upregulation of *serpinA3N* in N42 hypothalamic neurons confirming the neuronal regulation of *serpinA3N* by leptin. Nonetheless, circulating leptin levels do not appear to be a major regulator of *serpinA3N* gene expression in the fasting and refed states as the regulation or *serpinA3N* occurs in opposition to circulating leptin levels, with fasting, which causes leptin levels to drop, increasing *serpinA3N* gene expression, while refeeding, which increases circulating leptin levels [51], results in decreased *serpinA3N* gene expression. However, this may be explained by the stimulation of *serpinA3N* gene expression by the increased levels of circulating free fatty acids which occur during fasting [52].

Leptin insensitivity, induced by a HFD, is well documented [22]*.* While this concept was not specifically tested in the present study, the transcriptomics data which combined leptin challenge on a LFD and HFD indicated that the effect of diet was separate from that of leptin challenge and that there was no interaction between the two indicating that upregulation of *serpinA3N* gene expression by leptin is unaffected by HFD; however, this remains to be specifically tested.

Increased time on experiment or age of the mice also appears to be a factor in the upregulation of *serpinA3N* gene expression in the ARC, VMH and DMH of the hypothalamus. Although the effects of extreme ageing were not investigated in the present study, the mice were older at the end of both the transcriptomics and the in situ hybridisation studies on HFD than at the beginning and both showed a clear effect on *serpinA3N* gene expression with time despite being measured by different approaches. The mice exposed to the HFD for the longest period of time varied in age between 12 weeks at the beginning of the study and 28 weeks at the end on the study. Twelve weeks of age corresponds to a young mature adult, and 28 weeks corresponds to early middle age with mice showing some signs of age-related changes [53]. Ageing has also been reported to induce inflammation in the hypothalamus via the IKKβ/NFκB pathway. Indeed, hypothalamic inflammatory changes have shown to control systemic ageing demonstrating the importance of this phenomenon to the health of the whole organism [54].

## Conclusions

We have shown the upregulation of a novel hypothalamic gene, *serpinA3N*, by HFD and leptin. Increasing age and fasting also resulted in an upregulation although to a lesser extent. The upregulation of *serpinA3N* expression by leptin and palmitic acid was confirmed in N42 neurones in culture along with the upregulation of *IL-6* and *TNFα*, two classical markers of inflammation. Blocking the NFκB inflammatory pathway by BAY11 not only prevented the upregulation of *IL-6* and *TNFα*, but also that of *serpinA3N.* The protein product of *serpinA3N*, α_1_AC, is found in the media from cultured N42 neurons but is difficult to visualise within the cell lysate indicating that α_1_AC is a secreted protein in the hypothalamus as it is in the periphery.

These data strongly support the contention that *serpinA3N* expression is a marker of inflammation in the hypothalamus regulated by nutritional status and leptin and indicate a potential role for α_1_AC in hypothalamic inflammation and the regulation of energy balance.

## Methods

### Animal studies

All experiments were carried out according to the institutional and national guidelines of the European Convention of Vertebrate Animals Used for Experimentation, under European Council Directive 86/609/EEC dated November 1986. For the transcriptomics studies, male, 16-week-old, C57BL/6J mice were obtained from Charles River laboratories (Maastricht, The Netherlands) and housed in pairs in the light- and temperature-controlled animal facility of Wageningen University (12:12-h light/dark cycle at 22 °C). Mice were maintained for 1 or 4 weeks on either a HFD (45% kCal from fat) or LFD (10% kCal from fat) (Research Diet Services BV, Wijk bij Duurstede, The Netherlands) modelled on (D12451 and D12450B respectively Research Diets, NJ, USA). (Please see https://researchdiets.com/opensource-diets/dio-series-diets for diet composition details). For this study, palm oil was used in place of lard. They were then challenged with three intraperitoneal (IP) injections of murine leptin (2 mg/kg body weight) (R&D Systems, Abingdon, UK) or carrier at 24, 15 and approximately 3 h prior to killing in the early morning (*n* = 10). This experiment was part of the NuGO Proof of Principle studies as previously described [55].

To further investigate the regulation *of serpinA3N* gene expression on a HFD, male C57BL/6J mice (Harlan, Bicester, UK), aged 12 weeks, were singly housed on grid floors and fed either a HFD (60% kCal from fat) or a LFD (10% kCal from fat) (D12452 and D12450B respectively, Research Diets, NJ, USA—see web site above for detailed dietary breakdown), ad libitum for 3 days, 1, 4, 8, 12 or 16 weeks and killed either by exsanguination or by perfusion fixation (1 week) with 4% paraformaldehyde in phosphate buffered saline (PBS) under terminal anaesthesia (*n* = 6). To further investigate the upregulation of *serpinA3N* by leptin, male mice on the C57BL/6J background either leptin deficient (*ob/ob*) or lacking functional leptin receptors (*db/db*) and their lean littermates (Harlan, Bicester, UK) were killed for in situ hybridisation (*n* = 6). Leptin-deficient *ob/ob* mice and lean littermates (*n* = 6) were also challenged with vehicle or leptin by intraperitoneal (IP) injection (2 mg/kg body weight) and killed either 1 or 4 h later as detailed above. Food intake in response to leptin was not measured at this time because any changes were deemed to be impractical to measure with any accuracy due to the fact that leptin challenge was carried out over such a short period of time during the light period when food intake is already low. To confirm the upregulation of *serpinA3N* expression by HFD is independent from that of leptin, *ob/ob* mice were fed either a LFD 10% kCal from fat or a HFD either 45% or 60% kCal from fat in the form of lard (diets detailed above) for 8 weeks prior to killing by exsanguination.

To test the effect of nutritional status on hypothalamic *serpinA3N* gene expression, C57BL/6J mice were fasted for 24 h or fed ad libitum on standard mouse diet prior to killing. Refed animals were allowed access to food ad libitum for a period of 24 h after fasting (*n* = 8).

To test the possibility that inflammation plays a role in the effect of HFD on the expression of *serpinA3N*, mice from *IL-1R1*^*−/−*^ breeding pairs, on C57BL/6J background, purchased from Jackson laboratories were bred at University College Dublin (UCD) for 6–10 generations under specific pathogen-free conditions. Male IL-1R1^−/−^ mice were then fed HFD, 45% kCal or LFD, 10% kCal from fat for 12 weeks prior to killing (D12451 and D12450B respectively, Research Diets, NJ, USA). In this study, palm oil was used instead of lard (*n* = 9 HFD and *n* = 5 chow).

### Transcriptomics

Hypothalami were dissected and snap-frozen in liquid nitrogen and stored at − 80 °C until RNA extraction. Total RNA was extracted using TRIzol reagent (Invitrogen, Carlsbad, CA), and the mRNA purified with RNeasy Mini Kit (Qiagen, Venlo, The Netherlands) with 1% β-mercapto ethanol and treated with DNAse. The mRNA integrity was checked on an Agilent 2100 Bioanalyzer (Agilent Technologies, Amsterdam, The Netherlands) (average RIN 9.2), and individual RNA samples (4 μg per animal) were labelled and hybridised on a Affymetrix NuGO mouse array as described previously [35].

### In situ hybridisation

The riboprobe for *serpinA3N*, accession no. BC013651, was derived using the forward primer 5′CTACGCGGGCAAGAGGA3′ and the reverse primer 5′AAGGGGGCAATTTCAGTTT3′. The size of the amplified insert was 543 bp. Automated sequencing was performed to verify the probe sequence.

Messenger RNA levels were quantified by in situ hybridisation, on 20-μm thick coronal hypothalamic cryo-sections, using techniques described in detail elsewhere [56]. Briefly, slides were fixed in 4% (*w*/*v*) paraformaldehyde in 0.1 mol/l PBS for 20 min at room temperature, washed in PBS, incubated in 0.1 mmol/l triethanolamine for 2 min and acetylated in 0.1 mmol/l triethanolamine and 0.25% (*v*/*v*) acetic anhydride for 10 min. Sections were dehydrated in ethanol and dried under vacuum before hybridisation with riboprobes at 10^6^ cpm/ml for 18 h at 58 °C. After hybridisation, sections were desalted through a series of washes in standard saline citrate (SSC) to a final stringency of 0.1× SSC at 60 °C for 30 min, treated with RNase A and dehydrated in ethanol. Slides were apposed to Biomax MR (Sigma-Aldrich, UK) together with [^14^C] micro-scale standards (Amersham International, Amersham, UK) at room temperature for varying lengths of time depending on the probes used to ensure that the optical density of areas to be measured fall within the linear region of the standard curve.

Autoradiographs were scanned on a Umax Power Look II (UMAX Data Systems, Fremont, CA, USA). Integrated optical densities (IOD) of specific nuclei were quantified with reference to a mouse brain atlas [57] using the Image Pro-plus system (Media Cybernetics, Silver Springs, MD, USA). IOD was converted to nCi/g using [^14^C] microscale standard curves for measures of total gene expression.

### Immunohistochemistry

Frozen sections (20 μm) were first incubated with goat anti-mouse serpinA3N (R&D Systems cat. no. AF4709) at a dilution of 1:1000 overnight at 4 °C then incubated for 30 min at room temperature with biotinylated anti-goat IgG (Vector Labs cat. no. PK-6105) according to the manufacturer’s instructions followed by ABC reagent (Vector Labs cat. no. PK-6105) and stained with 3,3′-diaminobenzidine (DAB) substrate solution (brown) (Vector Labs cat. no. SK-4100). Sections were then thoroughly washed and incubated with either chicken anti-GFAP (Millipore, UK cat. no. AB5541), rabbit anti-IbA1 (Wako cat. no. 019-19741), rabbit anti-NPY (ThermoFisher Scientific cat. no. PA5-19568) or rabbit anti-AgRP (Phoenix Pharmaceuticals cat. no. H-003-57) at a dilution of 1:300 overnight at 4 °C and then at room temperature for 30 min with either goat anti-chicken IgG (Vector Labs cat. no. BA-9010) at a dilution of 1:200 followed by Vectastain Elite ABC kit (goat IgG) (Vector Labs cat. no. PK-6105) or directly with the Vectastain Elite ABC kit (goat IgG) and stained with Vector SG (blue) (Vector Labs cat. no. SK-4700). Control sections omitted the primary antibodies.

### Neuronal culture and reagents

To investigate the regulation of *serpinA3N* gene expression specifically in neurons, the embryonic immortalised hypothalamic cell line, N42, was used (Cellution Biosystems Inc., Ontario, Canada. Please see https://www.cedarlanelabs.com/Products/Detail/CLU122 for a comprehensive list of all genes expressed by these cells including the leptin receptor). These cells have been previously shown to provide a good model for hypothalamic neuron gene expression studies [44].

Cells were cultured in Dulbecco’s modified Eagle’s medium (DMEM) (Life Technologies, Paisley, UK) supplemented with 10% fetal bovine serum and 1% penicillin/streptomycin (Life Technologies) maintained at 37 °C under 5% CO_2_. Neurons were grown in 60-mm plates (*n* = 6) to 90% confluence then challenged with either 50 nM leptin (R&D Systems, Abingdon, UK), 200 μM palmitic acid or 200 μM oleic acid conjugated to fatty acid-free bovine serum albumin (BSA) (Sigma-Aldrich, UK), or the appropriate vehicle as a control. For leptin, this was a mixture of 12 μM HCl and 6 μM NaOH and for fatty acids 50 μM fatty acid-free BSA. To inhibit the NFkB pathway, N42 neurons were treated with 50 μM BSA or 200 μM palmitate in the presence and absence of 25 μM of the NFkB inhibitor BAY 11-7082 (Cayman Chemical, UK). All solutions were filter sterilised prior to use. For all treatments, medium was removed after 6 h.

### Real-time PCR

After the challenge, the medium was removed and cells were lysed using RLT lysis buffer (RNeasy Mini Kit Qiagen, Venlo, The Netherlands). Cell lysates were then collected and RNA extracted using an RNeasy Mini Kit (Qiagen) following the manufacturer’s instructions. RNA integrity was assessed by an Agilent 2100 Bioanalyzer (Agilent Technologies, Amsterdam, The Netherlands). One microgram of total RNA was reverse transcribed to generate cDNA using Superscript II reverse transcriptase (Life Technologies, CA, USA). Real-time PCR was carried out using Taqman Fast Universal PCR master mix (Applied Biosystems, USA) according to manufacturer’s instructions using the following Taqman assays: *B2M* (Mm00437762_m1), *serpinA3N* (Mm00776439_m1), *IL-6* (Mm00446190_m1) and *TNFα* (Mm00443258_m1). Each reaction used 1 μL of cDNA template.

### Western blotting

N42 neurons were grown as described earlier. The cell culture medium was collected and protein concentrated using an Amicon Ultra-0.5 Centrifugal Filter (10 K device, Millipore, UK). For lysate preparation, cells were scraped into 1 ml of PBS and pelleted by centrifugation; 1 ml of M-PER mammalian protein extraction reagent (Thermo scientific) was added to the cell pellet before sonication using a Sanyo Soniprep 1500 to ensure complete cell lysis. Protein concentrations were determined using the Pierce 660 nm protein assay reagent (Thermo Scientific). Samples were then diluted 3:1 with 4× Laemmli sample buffer (Bio-Rad, UK) with 2-mercaptoethanol (9:1 ratio) and boiled for 10 min, and 2 μg of protein was loaded on the gels. For media in which no cells were cultured, used as a control, an amount equivalent to 5 μL of concentrated media was loaded and as a positive control 12.5 ng of recombinant mouse serpin α_1_AC protein (R&D Systems). Proteins were separated on 10% mini-PROTEANTGX Precast Gels (Bio-Rad) and transferred to a PVDF Membrane (Bio-Rad) before immuno-detection using goat anti-mouse serpinA3N (R&D Systems) and peroxidase-linked secondary antibody (Donkey polyclonal antibody to goat IgG (HRP), abcam, UK). Chemiluminescent signal on the blots was detected using a Fujifilm LAS-3000 Imager after incubation with Clarity Western ECL substrate kit (Bio-Rad) using the manufacturer’s recommended protocol. Additional gels were run in parallel and stained with Colloidal Coomassie Blue (Safe Stain) (Severn Biotech Ltd) in order to visualise total protein present in cell media and lysates.

### Statistical analysis

Data are presented as mean ± SEM and were analysed using GenStat (GenStat, Eighth Edition (2005), VSN International Ltd., Oxford). In the case of experiments testing the influence of a single factor, a one-way ANOVA was performed. Where two or three factors were compared in a single experiment, a two- or three-way ANOVA followed by post hoc Student’s *t* tests based on the LSD were performed. In this case, the ANOVA results are expressed in the figure legend and the results of the Student *t* test are represented on the graph. *P* < 0.05 was considered statistically significant.

## Abbreviations

AgRP: Agouti-related peptide
ANOVA: Analysis of variance
ARC: Arcuate nuclei
BSA: Bovine serum albumin
CART: Cocaine- and amphetamine-regulated transcript
*db/db*: Diabetic obese mice with a spontaneous non-functional mutation in the leptin receptor
DMEM: Dulbecco’s modified Eagle’s medium
DMH: Dorsomedial nuclei of the hypothalamus
GFAP: Glial fibrillary acidic protein
HFD: High-fat diet
Iba1: Ionised calcium binding adaptor molecule 1
IKKβ: Inhibitor of nuclear factor kappa-B kinase subunit β
IL-1: Interleukin-1
*IL-1R1*^*−/−*^: Interleukin-1 receptor knockout mice
IL-6: Interleukin-6
IOD: Integrated optical density
IP: Intraperitoneal
JNK: c-Jun amino-terminal kinase
kCal: Kilocalories
LFD: Low-fat diet
LH: Lateral hypothalamus
LSD: Least significant difference
N42: mHypoE-N42 hypothalamic neuronal cell line
NFκB: Nuclear factor kappa-light-chain-enhancer of activated B cells
NPY: Neuropeptide Y
NuGO: Nutrigenomics Organization
*ob/ob*: Obese mice with a spontaneous mutation in the gene which produces leptin
PBS: Phosphate-buffered saline
POMC: Proopiomelanocortin
SEM: Standard error of the mean
SSC: Standard saline citrate
TLR4: Toll-like receptor 4
*TNFα*: Tumor necrosis factor alpha
UCD: University College Dublin
VMH: Ventromedial nuclei of the hypothalamus
α_1_AC: Alpha 1-antichymotrypsin
